# The endothelial activation and stress index (EASIX) predicts hazards of short- and long-term mortality in acute ischemic stroke: a retrospective cohort study

**DOI:** 10.1007/s00415-026-13941-8

**Published:** 2026-06-22

**Authors:** Antonia Kleeberg, Thomas Luft, Peter A. Ringleb, Julian Hotz, Lisa Kaindl, Marek Sykora, Daniel Golkowski, Jan C. Purrucker

**Affiliations:** 1 Department of Neurology, Heidelberg University Hospital, Im Neuenheimer Feld 400, 69120 Heidelberg, Germany; 2 Department of Internal Medicine V, Hematology, Oncology and Rheumatology, Heidelberg University Hospital, Heidelberg, Germany; 3 Department of Neurology, Hospital of St. John of God, Vienna, Austria; 4 Department of Neurology, University Hospital Bern, Bern, Switzerland; 5 Medical Faculty, Sigmund Freud University, Vienna, Austria

**Keywords:** Ischemic stroke, Endothelium, Biomarkers, EASIX, Endothelial dysfunction

## Abstract

**Introduction:**

Endothelial activation and stress index (EASIX) is a biomarker of endothelial dysfunction and has been validated previously as a prognostic score for mortality in various diseases, including oncologic diseases, sepsis, and cardiac disease. Since endothelial dysfunction is an established mediator of adverse outcomes in acute ischemic stroke, this study investigates the prognostic value of EASIX for risk of mortality in these patients.

**Patients and methods:**

We analyzed data from the Heidelberg (n = 4,188) and Vienna (n = 2,273) prospective acute ischemic stroke registries. EASIX was calculated as creatinine [mg/dL] × LDH [U/L] / platelet count [10^9^/L]. An EASIX cut-off was established using maximal Youden index. Validation was performed using Brier score and C-statistics.

**Results:**

Higher EASIX was associated with higher risk of mortality in the training cohort in a multivariable Cox regression (HR of all-cause mortality per log2 increase: 1.20 (95% CI 1.12–1.28), *p* < 0.001). An optimal EASIX cut-off value of 1.211 was identified in the derivation cohort. In the independent validation cohort, this cut-off was associated with risk of 3-month mortality in a multivariable binary logistic regression model (OR 1.86 (1.28–2.70), *p* < 0.01). Brier score and C-statistics validated the superior predictive performance of EASIX in the multivariable model.

**Discussion and Conclusion:**

EASIX predicts mortality in acute ischemic stroke patients and retained prognostic validity across two heterogeneous European cohorts. Incorporation of EASIX improved risk stratification beyond established clinical scores. EASIX may serve as a useful tool for risk stratification and outcome prediction in acute ischemic stroke patients.

**Supplementary Information:**

The online version contains supplementary material available at 10.1007/s00415-026-13941-8.

## Introduction

Despite major advances in acute stroke care, including intravenous thrombolysis (IVT) and endovascular therapy (EVT), mortality after acute ischemic stroke remains substantial [1]. Early identification of patients at high risk of death is crucial for informed clinical decision-making, risk stratification, and efficient resource allocation. Most existing prognostic models rely predominantly on clinical and radiological parameters [2–4], but do not account for the vascular pathophysiology that critically contributes to stroke-related mortality. In addition, many scores developed to predict functional outcome or mortality in acute ischemic stroke have limited clinical applicability as they are complex, incorporate numerous variables [5], and are not recommended by current national or international guidelines. Against this background, biomarkers reflecting endothelial injury—such as the Endothelial Activation and Stress Index (EASIX) [6], may provide complementary and clinically meaningful prognostic information.

Endothelial dysfunction represents a central mechanism in both the development and outcome of stroke and is characterized by impaired vasodilatory capacity, a prothrombotic state, inflammatory activation, and loss of vascular homeostasis.[7, 8] It can be triggered by activation of the renin–angiotensin–aldosterone system, oxidative stress through the formation of reactive oxygen species, or shear stress, with cardiovascular risk factors being well-established contributors [7, 9].

Recent evidence indicates that specific markers of endothelial dysfunction, including von Willebrand factor, soluble E-selectin, and soluble intercellular/vascular cell adhesion molecule 1 [10], are associated with clinical outcomes following endovascular thrombectomy for acute ischemic stroke, independent of traditional patient characteristics. While these mechanisms highlight the importance of endothelial dysfunction in stroke pathophysiology, such markers have limited applicability in routine clinical practice.

In hematologic and oncological research, EASIX was developed as a simple and readily available marker of endothelial dysfunction [6]. EASIX is calculated as *(creatinine* × *lactate dehydrogenase)/platelet count*, with each component reflecting distinct aspects of endothelial injury: creatinine as an indicator of renal endothelial impairment, lactate dehydrogenase (LDH) as a marker of cellular turnover and endothelial activation, and platelet count reflecting consumption in procoagulant states [11, 12]. Unlike conventional endothelial biomarkers, which provide information limited to specific vascular compartments and exhibit substantial spatial and temporal variability, EASIX correlates with glycocalyx thickness in sublingual vessels, digital perfusion, and platelet aggregation. It therefore appears to capture systemic responses to endothelial stress [13]. Consequently, EASIX may be more stable and reproducible compared to other biomarkers, as observed in patients with coronary artery disease two months before and six months after cardiac intervention [14].

Building on its role as a marker of systemic endothelial dysfunction, EASIX has demonstrated prognostic value in predicting mortality across a broad range of oncological and non-oncological conditions, including sepsis, COVID-19, preeclampsia, and traumatic brain injury [15–24]. Elevated EASIX scores have also been linked to cardiovascular comorbidities [15], consistent with evidence demonstrating its prognostic relevance in patients with cardiac disease [25, 26]. In patients with self-reported stroke history—without detailed information on stroke type or severity—EASIX was associated with stroke prevalence, all-cause and cardiovascular mortality [27]. In Asian and American populations, EASIX has been associated with increased mortality among patients with acute ischemic stroke admitted to intensive care units or treated with thrombectomy. [28, 29] To date, only one study has examined the association of EASIX with outcome following acute ischemic stroke in a European population, reporting a potential association specifically within the small-vessel disease subgroup [30].

Given the prognostic value of EASIX across diverse clinical settings and the well-established role of endothelial dysfunction in stroke, [8] we hypothesized that EASIX may serve as an independent predictor of mortality in patients with acute ischemic stroke.

## Patients and methods

### Study design and population

For this investigator-initiated, retrospective cohort study, we collected data from two independent cohorts of patients with acute ischemic stroke. The derivation cohort consisted of patients who were treated with acute recanalization therapies (IVT and/or EVT) at Heidelberg University Hospital between January 2015 and June 2024. For the validation cohort, patients treated at St. John’s Hospital, Vienna, Austria between May 2018 and December 2024 were included regardless of whether they received acute recanalization therapies. The cohorts represent independent populations treated during different time periods, reflecting evolving clinical practices and center-specific protocols. Neither age > 80 years nor pre-stroke disability constituted exclusion criteria for receiving recanalization therapies. For patients with multiple ischemic strokes within the observation period, only the first qualifying event was analyzed in the derivation cohort. Patients aged < 18 years were excluded. Additionally, patients were excluded if one laboratory parameter required for EASIX calculation was missing or—in the derivation cohort—if follow-up data on survival were unavailable. Finally, patients with pre-existing hemodialysis dependence were excluded in the derivation cohort owing to potential iatrogenic alterations in laboratory parameters that could confound EASIX calculations (no dialysis data were available for the validation cohort) [31–33]. Patient flow is illustrated in Supplementary Figure S1 (derivation cohort) and Supplementary Figure S2 (validation cohort).

### Data acquisition and outcome measures

Patient data for the derivation cohort were obtained from the Heidelberg prospective recanalization database (HeiReKa), the central laboratory, and the hospital information system. Survival data for the derivation cohort were retrieved through automated queries to the municipal registration office. Survival status was assessed as of October 25, 2024. In the validation cohort, only 3-month outcomes were available. The validation cohort included consecutive patients from a prospective comprehensive stroke center database at the St. John’s Hospital, Vienna, Austria.

Neurological status at admission was assessed using the National Institutes of Health Stroke Scale (NIHSS). Functional outcomes before and after stroke were assessed using the modified Rankin scale (mRS; range 0 [no symptoms] to 6 [death]). 3-month follow-up outcomes were determined through outpatient assessments, standardized interviews, or rehabilitation facility reports. All follow-up data were systematically integrated into a prospective database to ensure completeness and consistency. Baseline Alberta Stroke Program Early CT Score (ASPECTS) was assessed using visual ratings by board-certified neuroradiologists or, when appropriate, electronically calculated e-ASPECTS (Brainomix®). EASIX was calculated as (creatinine [mg/dL] x lactate dehydrogenase [LDH; U/L])/platelet count [10^9^/L]. Laboratory parameters for EASIX calculation were obtained from the first available measurement within 24 h of hospital admission. The study protocol was approved by the Ethics Committee at the Medical Faculty of the University Heidelberg (S-533/2024). This study was performed in accordance with the Strengthening the Reporting of Observational Studies in Epidemiology (STROBE) guidelines.

### Statistical analysis

Descriptive statistics were used to summarize demographic, clinical, and radiological characteristics. Categorical variables are presented as counts and percentages, whereas continuous variables are reported as mean (standard deviation) or median (interquartile range), depending on their distribution. Overall survival (OS) was defined as the interval from ischemic stroke onset to death from any cause. Time-to-event data were illustrated using Kaplan–Meier survival curves. Comparisons of baseline characteristics and outcomes between groups were performed using Mann–Whitney U test for continuous variables and chi-square test for categorical variables.

Multivariable Cox proportional hazards regression was applied to identify predictors of survival in the derivation cohort where long-term outcome data were available. The initial full model included the following covariates: age, sex, pre-stroke functional status, NIHSS at admission, relevant medical history, and recanalization therapies (IVT and/or EVT) (see Supplementary Table S3 for full list of variables). Variables with p < 0.05 in the full model were retained in a final reduced model. Subgroup analysis was conducted by incorporating interaction terms within the Cox model. Multicollinearity among predictors was assessed by calculating the variance inflation factor and tolerance.

Binary outcome data (3-month survival) were analyzed with a binary logistic regression incorporating the variables from the full Cox model that were available in both the derivation and validation cohorts. Both Cox and logistic regression analyses utilized the log2-transformed endothelial activation and stress index (log_2_(EASIX)) as previously described, [6] to meet linearity assumptions of the regression models. The distribution of log_2_(EASIX) was visually assessed through quantile–quantile plots and histograms [6].

All regression analyses were performed as complete case analyses without imputation of missing values. Given the relatively high proportion of missing 3-month outcome data in the validation cohort, best- and worst-case scenarios were evaluated as a sensitivity analysis.

Participants were stratified into quartiles based on the 25th, 50th, 75th, and 100th percentiles of log_2_(EASIX), or according to quartiles of a combined prognostic index (PI). In the derivation cohort, a PI was calculated following Royston et al. [34] as the linear combination of regression coefficients and individual values of the variables (PI = Σβ_i_ × X_i_). This continuous PI was calculated with and without log_2_(EASIX) and was log-transformed to calculate the respective probabilities. Model performance was assessed using Brier scores for three approaches: log_2_(EASIX) alone, a PI including log_2_(EASIX), and a PI without log_2_(EASIX). Brier score reflects the mean square error of misspecification between predicted probabilities and the true outcome, respectively, with lower values indicating better model calibration and discrimination (range 0–1, where 0 represents perfect prediction). [35] Discriminatory performance of the binary logistic regression model was evaluated using the C-statistics (concordance statistics), equivalent to the area under the receiver operating characteristic (ROC) curve for binary outcomes [36]. For external validation, PI coefficients derived from the derivation cohort were applied to the validation cohort to calculate Brier scores and C-statistics. The optimal cut-off value was determined by ROC analysis using maximization of the Youden index in the derivation cohort. [37]

No a priori sample size calculations were performed as this was an exploratory analysis using all available registry data. Statistical analysis was conducted using Microsoft Excel® (version 2411, Microsoft Corporation, Redmond, WA, USA), SPSS Statistics® (version 30, IBM Corporation, Armonk, NY, USA), GraphPad Prism (version 10.4.2, GraphPad Software, San Diego, CA, USA), R statistical software (v4.4.3, R core team 2025) with the “Evaluate Cutpoints” package[38] and Matlab (R2024b, The MathWorks, Inc., Natick, MA, USA). Two-sided *p* values < 0.05 were considered statistically significant.

## Results

### EASIX independently predicts hazard of mortality

Of 5,213 stroke patients treated with recanalization therapies, 4,188 were included in the final analysis of the derivation cohort. Median age was 76 years (IQR 66–83), 51.2% were female, median pre-stroke mRS was 0 (IQR 0–2), median NIHSS at admission was 12 (IQR 6–19), and median ASPECTS was 10 (IQR 8–10). IVT was performed in 60.8% of patients, EVT in 72.7%, and both therapies in 33.5%. Baseline characteristics did not differ between excluded and included patients (Supplementary Table S1).

Outcome data are summarized in Supplementary Table S2. 3-month survival data were missing in *n* = 57 patients (1.36%). During a median follow-up of 25 months (IQR 3–58), 43.7% (*n* = 1832) of patients died. Median time to death was 1 month (IQR 0–22), with 22% 3-month mortality (*n* = 907/4131).

**Fig. 1:**
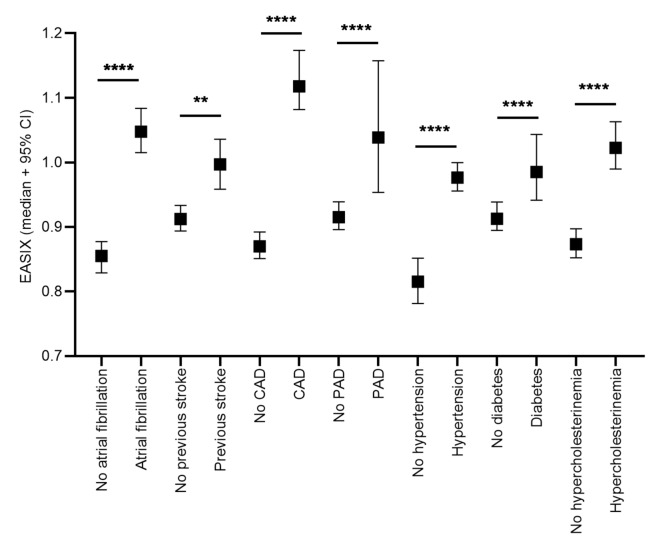
EASIX is significantly higher in patients with pre-existing cardiovascular diseases (derivation cohort). Two-tailed Mann–Whitney U test. Median and 95% confidence interval are shown. ** equals *p* < 0.01, **** equals *p* < 0.0001. *CAD* coronary artery disease, *PAD* peripheral artery disease

EASIX levels were significantly higher in patients with pre-existing cardiovascular disease, consistent across all individual cardiovascular subgroups (Fig. 1).

For identifying predictors of mortality, we employed Cox proportional hazards regression with all-cause mortality as the time-to-event endpoint, using a two-step approach. First, we constructed a comprehensive model incorporating all routinely available variables (*n* = 23), including premorbid conditions and stroke characteristics (Supplementary Table S3). No multicollinearity was detected (Supplementary Table S4). Variables with *p* < 0.05 (*n* = 14) were subsequently entered into a restricted model. Log_2_(EASIX) emerged as a robust independent predictor of risk of mortality (HR = 1.20, 95% CI 1.12–1.28, *p* < 0.01, Supplementary Table S5), indicating that each doubling of EASIX corresponded to a 20% increased hazard of death. Other significant predictors with comparable effect sizes included sex (HR = 1.30, 95% CI 1.16–1.45 *p* < 0.01), pre-stroke mRS (HR = 1.26, 95% CI 1.21–1.32, *p* < 0.01), previous diabetes (HR 1.42, 95% CI 1.26–1.60, *p* < 0.01), previous anticoagulation (HR = 1.22, 95% CI 1.05–1.40, *p* < 0.01), previous antiplatelet therapy (HR = 1.22, 95% CI 1.08–1.38, *p* < 0.01), and IVT treatment (HR = 0.77, 95% CI 0.69–0.87, *p* < 0.01).

**Fig. 2:**
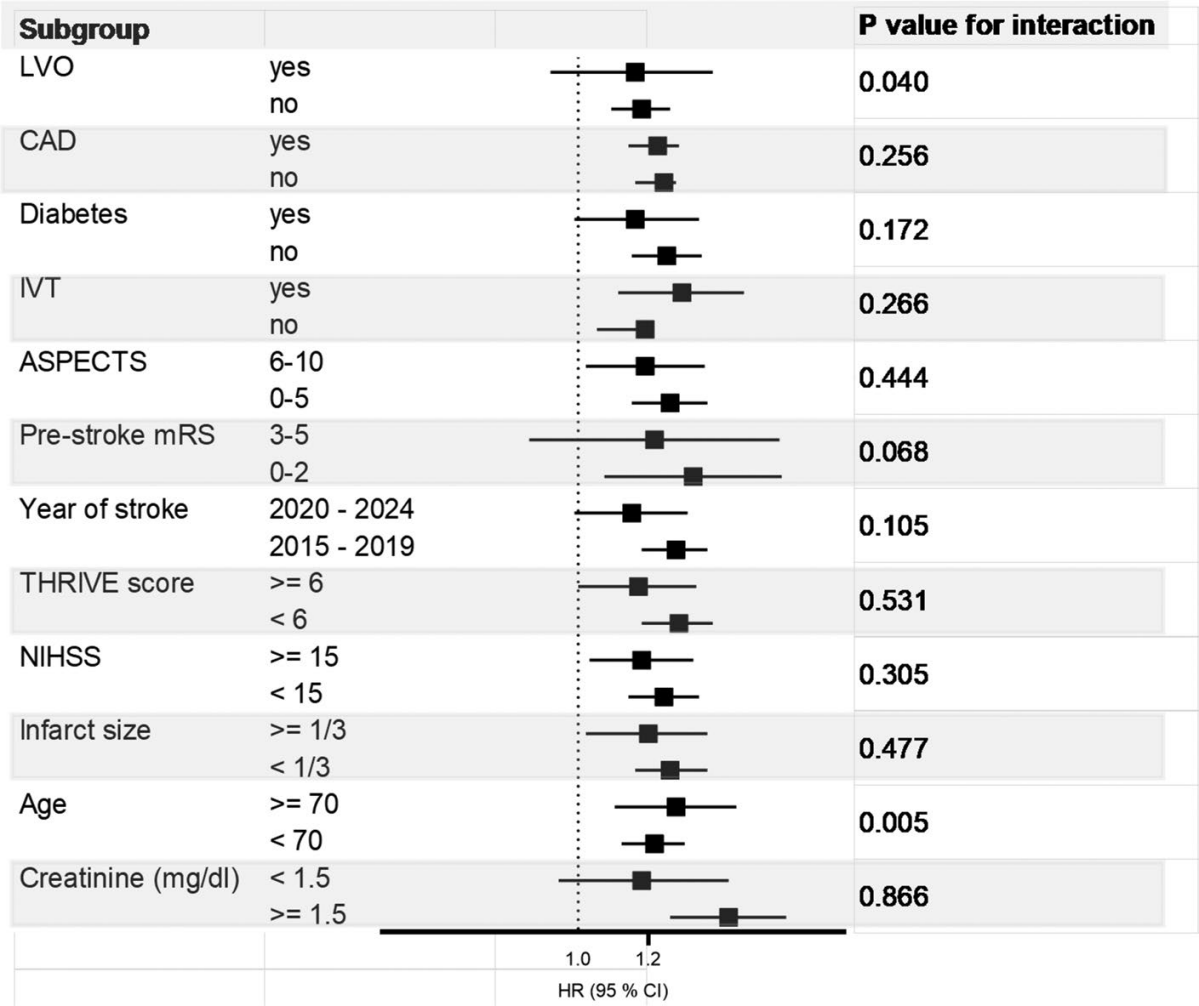
Forest plot for subgroup analysis of the association between log_2_(EASIX) and mortality in acute ischemic stroke patients (derivation cohort, Cox proportional hazards regression, restricted model, endpoint all-cause mortality). The trend of the effect size was consistent in all subgroups. Hazard ratio (HR) and 95% CI are shown. Significant interactions were found for age (*p* = 0.005) and presence of large vessel occlusion (*p* = 0.04). *P* values < 0.05 were considered significant, *LVO* large vessel occlusion. *CAD* coronary artery disease. *IVT* intravenous thrombolytic therapy, *ASPECTS* Alberta Stroke Program Early Computed Tomography Score, *mRS* modified Rankin Scale, *NIHSS* National Institutes of Health Stroke Scale, *Log*_*2*_*(EASIX)* log2-transformed Endothelial Activation and Stress Index. THRIVE (Totaled Health Risks in Vascular Events) score ranges from 0–9 points, calculated from age, baseline NIHSS, and the presence of hypertension, diabetes, and atrial fibrillation; higher scores indicate worse prognosis

Log_2_(EASIX) showed consistent associations with mortality risk across subgroups (Fig. 2; Supplementary Table S6), with consistent effect directions. Significant interactions occurred for age (*p* = 0.005) and large vessel occlusion (*p* = 0.04). In patients younger than 70 years, log_2_(EASIX) levels showed a stronger association with mortality risk (HR = 1.47, 95% CI 1.26–1.71) compared with those aged 70 years or older (HR 1.16, 95% CI 0.92–1.45; *p* for interaction < 0.01). Additional subgroup analysis stratified by stroke etiology according to the TOAST classification (Supplementary Figure S6) [39] demonstrated consistent effect directions across all subtypes, with the exception of small-vessel disease, in which log_2_(EASIX) showed no significant association with mortality risk. This finding should be interpreted with caution, however, as the small-vessel disease subgroup comprised only 33 events, raising concern for insufficient statistical power and potential model instability.

Each individual EASIX component was significantly associated with all-cause mortality, supporting their combined prognostic relevance in the composite index (Supplementary Table S7).

### External validation of EASIX’s predictive value

To validate EASIX’s prognostic value, we analyzed an independent external cohort from Vienna (*n* = 2,273). Patients in the validation cohort differed substantially from the derivation cohort in several characteristics: stroke severity (median NIHSS 4 vs. 12), rate of large vessel occlusions (24.4% vs. 83.0%), recanalization therapies (36.0% vs. 100.0%), and 3-month survival (mortality at 3 months 16.6% vs. 22.0%). These differences reflect different cohort designs: The derivation cohort comprised patients from the Heidelberg recanalization registry exclusively including patients treated with IVT and/or EVT, while the validation cohort from Vienna represented a comprehensive stroke registry. Long-term follow-up data were unavailable for the validation cohort. Baseline characteristics and outcomes are summarized in Tables 1 and 2; patient selection is illustrated in Supplementary Figure S2.

**Table 1:** Baseline characteristics in comparison between the derivation and the validation cohort

	Derivation cohort	Validation cohort	*P* value
All patients, n	4188	2273	
Demographics			
Female sex (n/N, %)	2146/4188 (51.2%)	1102/2273 (48.5%)	0.0341
Age (median [IQR])	76 (66–83) [*N* = 4188]	75 (64–82) [*N* = 2273]	< 0.01
Functional status			
Pre-stroke mRS (median [IQR])	0 (0–2) [N = 4150]	0 (0–2) [*N* = 2273]	< 0.01
NIHSS score at admission (median [IQR])	12 (6–19) [*N* = 4172]	4 (4–9) [*N* = 2259]	< 0.01
Acute stroke treatment			
Vessel occlusion in CT angiography (n/N, %)	3446/4152 (83.0%)	555/2273 (24.4%)	< 0.01
ASPECTS (median [IQR])	10 (8–10) [*N* = 3516]	na	na
Systolic blood pressure (median [IQR])	160 (141–175) [*N* = 3664]	na	na
IVT (n/N, %)	2547/4188 (60.8%)	664/2272 (29.2%)	< 0.01
EVT total (n/N, %)	3046/4188 (72.7%)	679/2237 (30.4%)	< 0.01
Intracranial EVT (n/N, %)	2710/4178 (64.9%)	na	na
Extracranial EVT (n/N, %)	495/4188 (11.8%)	na	na
EASIX (median [IQR])	0.928 (0.658–1.357)	0.836 (0.591–1.232)	< 0.01
Prior medication			
Mono-platelet inhibition (n/N, %)	1243/4130 (30.1%)	679/2273 (29.9%)	0.8513
Dual platelet inhibition (n/N, %)	118/4130 (2.9%)	36/2273 (1.6%)	< 0.01
Anticoagulation (n/N, %)	772/4146 (18.6%)	342/2273 (15.0%)	< 0.01
Intake of statin (n/N, %)	1341/4083 (32.8%)	na	na
Comorbidities			
Atrial fibrillation (n/N, %)	1682/4164 (40.4%)	647/2273 (28.5%)	< 0.01
Previous Stroke (n/N, %)	842/4166 (20.2%)	462/2204 (21.0%)	0.4800
Coronary artery disease (n/N, %)	1019/4148 (24.6%)	na	na
Peripheral artery disease (n/N, %)	293/4107 (7.1%)	na	na
Arterial hypertension (n/N, %)	3103/4174 (74.3%)	1937/2259 (85.7%)	< 0.01
Diabetes mellitus (n/N, %)	969/4176 (23.2%)	605/2247 (26.9%)	< 0.01
Hypercholesterolemia (n/N, %)	1552/4143 (37.5%)	1548/2205 (70.2%)	< 0.01
Active smoker (n/N, %)	571/4066 (14.0%)	621/2120 (29.3%)	< 0.01

*mRS* modified Rankin Scale. *NIHSS* National Institutes of Health Stroke Scale. CT computed tomography, *ASPECTS* Alberta Stroke Program Early Computed Tomography Score. *IVT* Intravenous Thrombolytic Therapy. *EVT* Endovascular Stroke Therapy. *EASIX* Endothelial Activation and Stress Index

Patients from the derivation and validation cohorts differed significantly in terms of stroke severity, frequency of large vessel occlusions, performance of IVT and EVT. Patients in the derivation cohort more often had atrial fibrillation while hypertension, hypercholesterolemia and smoking were more frequent in the validation cohort. EASIX was significantly higher in the derivation cohort. Differences between the groups were examined through chi-square and Mann–Whitney U tests, respectively. *P* values < 0.05 were considered significant

**Table 2:** Functional outcome and short-term mortality in comparison between the derivation and the validation cohort

Outcome	Derivation cohort	Validation cohort	*P* value
mRS at 3-month, median (IQR)	3 (1–5)	2 (0–4)	< 0.01
Death			
In house (n/N, %)	512/4176 (12.3%)	62/2273 (2.7%)	< 0.01
At 3-month (n/N, %)	907/4131 (22.0%)	292/1758 (16.6%)	< 0.01
At end of follow-up observation period (n/N, %)	1832/4188 (43.7%)	Na	na

*mRS* modified Rankin scale, *IQR* interquartile range

Given the less-severely affected patients, patients from the validation cohort showed a better neurological short-term outcome. Data on long-term outcome were not available in the validation cohort. Differences between the groups were examined through chi-square and Mann–Whitney U tests, respectively. *P* values < 0.05 were considered significant

Given the availability of only 3-month outcome data in Vienna, we constructed logistic regression models including variables present in both cohorts (*n* = 18). In the derivation cohort, log_2_(EASIX) was significantly associated with 3‑month mortality (OR 1.41, 95% CI 1.26–1.57, *p* < 0.01; Supplementary Table S8). Using a prognostic index derived from the derivation cohort’s coefficients, model performance was validated in the Vienna cohort using Brier score and C-statistics.

The multivariable model including log_2_(EASIX) demonstrated better performance in the validation cohort: integrated Brier score of 0.0888 vs. 0.0956 and C-statistics of 0.869 vs. 0.857 compared with the same model excluding EASIX (Table 3), confirming EASIX’s independent predictive value. A univariable model with log_2_(EASIX) yielded a Brier score of 0.1335 and a C-statistics of 0.658, demonstrating that EASIX alone provides predictive values.

**Table 3:** Validation of log_2_(EASIX) as an independent predictor of 3-month survival in acute ischemic stroke patients (validation cohort adjusted to PI coefficients derived from the multivariable model of the derivation cohort)

	Prognostic Index (PI) with log_2_(EASIX)	PI without log_2_(EASIX)	Only log_2_(EASIX)
Brier score	0.0888	0.0956	0.1335
C-statistics	0.869	0.857	0.658

*EASIX* Endothelial Activation and Stress Index, *C-statistics* concordance statistic

The prognostic index with a multivariable model (*n* = 18, variables as in Supplementary Table S8) including log_2_(EASIX) shows a lower Brier score and a higher C-statistics compared to a model without EASIX

EASIX = Endothelial Activation and Stress Index. C-statistics: concordance statistic.

### Establishment of an EASIX cut-off for mortality risk stratification

We aimed to define an EASIX cut-off to stratify patients by mortality risk. We performed ROC analysis for 3-month mortality in the derivation cohort and determined the optimal cut-off by maximizing Youden index (Supplementary Figs. S3 and S4). This yielded an optimal EASIX threshold value of 1.211. This cut-off value corresponded to the 68th percentile in the derivation cohort and the 73rd percentile in the validation cohort, suggesting robustness across cohorts.

**Fig. 3:**
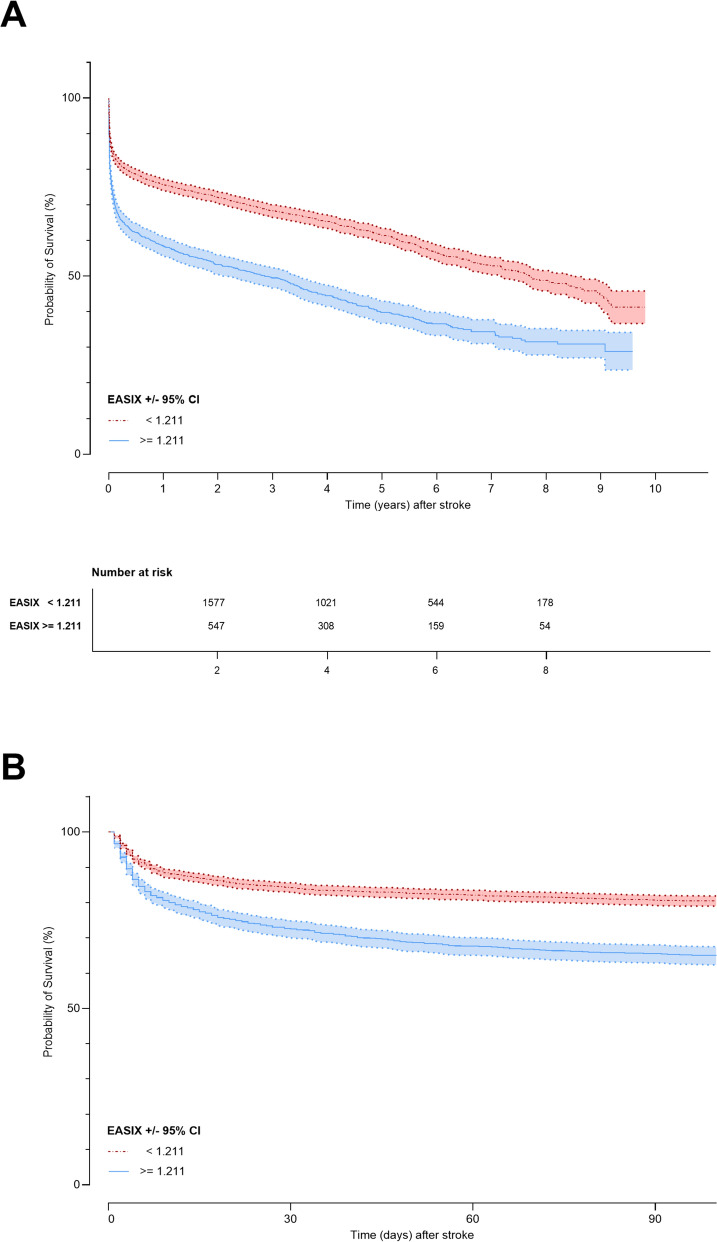
Survival analysis based on an EASIX cut-off value of 1.211. This Kaplan–Meier curve depicts graphically the differential survival depending on EASIX values, displayed through the newly established cut-off value of 1.211, including the depiction of 95% CI values. A log-rank test was performed to compare survival between groups. There was a statistically significant difference (χ^2^ = 160.636, *p* < 0.001). **A** Long-term survival analysis. **B** Short-term (90 days) survival analysis. *CI* confidence interval, *EASIX* Endothelial activation and stress index

When applied to the validation cohort, this threshold identified patients at increased 3-month mortality risk (OR 1.86, 95% CI 1.28–2.70, *p* < 0.01; Supplementary Table S9). Three-month mortality rates were 12.6% (163/1,291) in the low-risk group (EASIX < 1.211) compared to 27.6% (129/467) in the high-risk group (EASIX ≥ 1.211). In the derivation cohort, Kaplan–Meier analysis with log-rank testing confirmed this threshold’s long-term prognostic significance using follow-up data beyond 3 months (Fig. 3).

Again, using a prognostic index derived from the derivation cohort’s coefficients, this cut-off value was validated in the validation cohort using Brier score and C-statistic. The multivariable model including binary EASIX with cut-off 1.211 demonstrated better performance in the validation cohort: integrated Brier score of 0.0899 vs. 0.0956 and C-statistic of 0.867 vs. 0.857 compared with the same model excluding EASIX (Supplementary Table S10), confirming the predictive value of the previously established cut-off value. Detailed performance metrics for the derived cut-off value, including sensitivity, specificity, positive predictive value (PPV), and negative predictive value (NPV), are reported in Supplementary Table S18.

Best- and worst-case sensitivity analyses were performed to assess the robustness of the binary EASIX cut-off given the relatively high proportion of missing 3-month outcome data in the validation cohort and the different baseline characteristics between patients with and without follow-up information (Supplementary Tables S11 and S12). Both best- and worst-case logistic regression models confirmed that EASIX remained a significant predictor of mortality risk, with a stronger effect observed in the best-case scenario (Supplementary Tables S13 and S14).

These findings were consistent when evaluated using Brier score and C-statistics: Brier scores were lower and C-statistics higher in the best-case analysis, whereas the worst-case analysis showed effects in the same direction but of lesser magnitude than in the original cohort (Supplementary Table S15). The best-case scenario may approximate the true outcome as patients lacking 3-month data had milder strokes, younger age, and better premorbid status—factors consistently associated with a more favorable prognosis.

Functional outcome at 3 months differed according to EASIX levels. In the derivation cohort, patients with elevated EASIX values above the prognostic cut-off (≥ 1.211) had a 3-month mortality of 31.5%. In contrast, patients with low EASIX values (< 0.88), corresponding to the upper quartile of EASIX levels observed in healthy individuals from the EndoCDO-H study, [40] showed a 3-month mortality of 16.2% (Supplementary Figure S5).

As part of sensitivity analysis, we additionally examined the externally defined and validated EASIX cut-off value of 2.32 [14, 41]. When applied to the validation cohort in the multivariable model (*n* = 18 variables), this threshold identified patients at increased 3-month mortality risk with a similar effect size (OR 2.09, 95% CI 1.54–2.84, *p* < 0.01; data not shown). The resulting C-statistics, Brier score, sensitivity, specificity, PPV, and NPV in the validation cohort were comparable to those obtained with our newly established cut-off value (Supplementary Tables S18 and S19), thus providing further support for our findings.

### Added value of EASIX to previously established prognostic scores in acute ischemic stroke

Among established prognostic scores for stroke-related mortality and functional outcome, [5] the Totaled Health Risk in Vascular Events (THRIVE) score comprises five routinely available variables (age, NIHSS score, history of diabetes, hypertension and atrial fibrillation). To assess the incremental prognostic value of EASIX beyond the THRIVE score, binary EASIX was added to a logistic regression model including the THRIVE score components. In both the derivation and validation cohorts, inclusion of binary EASIX resulted in higher C-statistics compared with the model including the THRIVE score alone (Supplementary Table S16).

## Discussion

In this large, well-characterized cohort of recanalization-treated patients with acute ischemic stroke, we demonstrate that EASIX, a composite marker of endothelial dysfunction, is a strong and independent predictor of mortality risk. These findings were externally validated in an independent cohort from Vienna, with marked differences in stroke severity, treatment patterns, and other covariates. Importantly, the prognostic cut-off derived from the derivation cohort remained applicable in the external cohort, underscoring the robustness of EASIX among heterogenous stroke populations. To our knowledge, together with the paper by Makris et al. [30], this is one of the first studies systematically evaluating the prognostic relevance of EASIX in acute ischemic stroke patients within a European population.

Our findings extend prior observations from hematologic, oncologic, infectious, and critical care populations, where EASIX has consistently predicted adverse outcomes. [6, 15–22, 24, 42, 43] The present data reinforce the relevance of EASIX in cerebrovascular disease, a condition in which endothelial dysfunction is a key pathophysiological driver. [14, 26] Despite the heterogeneous etiologies of ischemic stroke—including atherosclerotic, cardioembolic, microangiopathic, and less-common inflammatory or hematologic causes—the consistent prognostic performance of a simple marker of systemic endothelial dysfunction is noteworthy. [13] EASIX has been linked to markers of endothelial homeostasis, such as angiopoietin 2, [15] interleukin 8 (CXCL8), interleukin 18, and insulin-like-growth-factor-1 (IGF-1), [13, 43, 44], and to functional indices of glycocalyx integrity and tissue perfusion, [13] supporting its biological plausibility. The fact that EASIX retained independent prognostic value across multivariable models—even after adjustment for established cardiovascular comorbidities and pre-stroke functional status as reflected by the modified Rankin Scale—argues against the notion that EASIX merely serves as a non-specific surrogate of general disease burden. EASIX’s prognostic impact has been independently validated in multiple clinical settings [45, 46].

In the Heidelberg derivation cohort, each doubling of EASIX was associated with a 20% increase in mortality risk, an effect size comparable to established clinical predictors, such as pre-stroke disability, diabetes, and sex. Other established predictors, including age, ASPECTS, pre-stroke mRS, and NIHSS also demonstrated expected associations [2, 3, 47, 48]. Markers of systemic inflammation, including C-reactive protein and leukocyte count, were associated with increased mortality, whereas statin pretreatment showed a protective association, in line with prior reports. The association between prior stroke and mortality likely reflects higher baseline morbidity, while the observed sex differences are consistent with known differences in life expectancy and comorbidity burden [47, 49, 50].

The inverse association between IVT and mortality contrasts with earlier reports of increased or unchanged short-term mortality [51, 52]. This finding most likely reflects confounding by indication, as the derivation cohort exclusively included patients undergoing recanalization therapy. Within this cohort, patients treated with EVT had a substantially higher stroke severity than those receiving IVT alone (see Supplementary Table S17), suggesting that treatment allocation primarily reflected stroke severity. Similarly, the association between prior antiplatelet therapy and increased mortality observed in the derivation cohort—but not in the validation cohort—likely reflects higher burden of vascular comorbidity among severely affected patients.

Beyond continuous risk estimation, we established an EASIX cut-off value that enabled clinically intuitive mortality risk stratification. This threshold demonstrated consistent prognostic performance across cohorts and remained robust in sensitivity analyses accounting for missing outcome data. Moreover, adding EASIX to the established THRIVE score improved model discrimination in both the derivation and validation cohorts, indicating that EASIX provides prognostic information beyond traditional clinical risk factors. Given the modest discriminatory performance observed for binary EASIX, it is not suitable as a stand-alone prognostic tool; however, our findings support the incremental value of incorporating a biomarker reflecting vascular pathophysiology into existing prognostic frameworks.

Several limitations merit consideration. First, the retrospective design introduces potential documentation bias. Second, exclusion of 630 patients (13.0%) from the derivation cohort and the exclusion of 515 patients (22.7%) from the validation cohort in the complete case analysis due to missing survival data may have introduced attrition bias. However, sensitivity analysis confirmed the robustness of the findings under both best- and worst-case assumptions despite differences in baseline characteristics. Third, individual components of EASIX can be influenced by factors unrelated to endothelial dysfunction, such as blood transfusions or contrast-induced acute kidney injury—a rare but recognized complication of endovascular stroke therapy [53]. Notably, sensitivity analysis stratified by creatinine level did not reveal any significant differences across subgroups, suggesting that the prognostic signal of EASIX is not primarily driven by renal impairment. Finally, long-term outcome data were unavailable for the external validation cohort, precluding assessment of EASIX’s long-term predictive performance in that population.

Despite these limitations, this study has several strengths, including a large, well-characterized derivation cohort, a rigorous two-step modeling strategy minimizing overfitting, and validation in an independent, broader stroke cohort with markedly different baseline characteristics. These features support the generalizability of EASIX as a prognostic marker across diverse clinical settings.

## Conclusion

EASIX represents a practical and biologically grounded prognostic tool for patients with acute ischemic stroke, offering added value beyond established clinical prognostic models. Its implementation could refine risk stratification, improve prognostic communication, and support clinical decision-making. In research settings, EASIX may further facilitate patient stratification in trials investigating endothelial-targeted interventions, including established therapies, such as statins, ACE inhibitors, isosorbide mono-nitrate, cilostazol, and SGLT2 inhibitors, as well as emerging approaches like endothelial progenitor cell therapy and vascular endothelial-cadherin stabilizers [54–59].

## Supplementary Information

Below is the link to the electronic supplementary material.

Supplementary file1 (DOCX 15610 KB)

## Data Availability

The data that support the findings of this study are available from the corresponding author upon reasonable request.
