# Supplementary material for: The endothelial activation and stress index (EASIX) predicts hazards of short- and long-term mortality in acute ischemic stroke: a retrospective cohort study
Source: J Neurol. 2026 Jun 22;273(7):413. doi: 10.1007/s00415-026-13941-8 (PMC13287146; doi:10.1007/s00415-026-13941-8)

**Supplementary materials**

**The Endothelial Activation and Stress Index (EASIX) predicts hazards of short- and long-term mortality in acute ischemic stroke: a retrospective cohort study**

Antonia Kleeberg, MD ^1^, Thomas Luft, MD, PhD ^2^, Peter A. Ringleb, MD ^1^, Julian Hotz, MD ^3,4^, Lisa Kaindl, MD ^3^, Marek Sykora, MD, PhD, MSc ^3,5^, Daniel Golkowski, MD ^1*^, Jan C. Purrucker, MD, MSc ^1*^

**Corresponding Author:**

Jan Purrucker, MD, Department of Neurology, Heidelberg University Hospital. E-mail: jan.purrucker@med.uni-heidelberg.de

**Supplementary Table S1: Baseline characteristics** (derivation cohort)
No significant differences were found in terms of baseline characteristics between included patients and patients in which EASIX could not be calculated or long-term survival data were missing. Differences between the groups were examined through Chi square and Mann Whitney U tests, respectively. P values < 0.05 were considered significant.

|  | **Patients included in final analyses** | **Patients in which EASIX values or long-term survival data were missing** | **P value** |
| --- | --- | --- | --- |
| All patients, n | 4188 | 739 |  |
| **Demographics** | |  |  |
| Female sex (n/N, %) | 2146/4188 (51.2 %) | 385/739 (52.1 %) | 0.67 |
| Age (median [IQR] | 76 (66-83) [N = 4188] | 77 (67-84) [N = 739] | 0.09 |
| **Functional status** | |  |  |
| pre-stroke mRS (median [IQR]) | 0 (0-2) [N = 4150] | 1 (0-2) [N = 726] | 0.31 |
| NIHSS score at admission (median [IQR]) | 12 (6-19) [N = 4172] | 12 (6-18) [N= 736] | 0.85 |
| **Acute stroke treatment** | |  |  |
| Vessel occlusion in CT-angiography (n/N, %) | 3446/4152 (83.0 %) | 616/729 (84.5 %) | 0.25 |
| ASPECTS (median [IQR]) | 10 (8-10) [N = 3516] | 9 (8-10) [N = 627] | 0.52 |
| Systolic blood pressure (median [IQR]) | 160 (141-175) [N = 3664] | 160 (143-175) [N=620] | 0.57 |
| IVT (n/N, %) | 2547/4188 (60.8 %) | 445/739 (60.2 %) | 0.76 |
| EVT total (n/N, %) | 3046/4188 (72.7 %) | 535/739 (72.4 %) | 0.85 |
| - Intracranial EVT (n/N, %) | 2710/4178 (64.9 %) | 471/737 (63.9 %) |  |
| - Extracranial EVT (n/N, %) | 495/4188 (11.8 %) | 90/739 (12.2 %) |  |
|  | **Patients included in final analyses** | **Patients in which EASIX values or long-term survival data were missing** | **P value** |
| Log_2_(EASIX) (median [IQR]) | -0.108 (-0.604-0.441) | -0.108 (-0.593-0.433) | 0.82 |
| **Prior medication** | |  |  |
| Mono platelet inhibition (n/N, %) | 1243/4130 (30.1 %) | 214/719 (29.8 %) | 0.61 |
| Dual platelet inhibition (n/N, %) | 118/4130 (2.9 %) | 16/719 (1.7 %) |  |
| Anticoagulation (n/N, %) | 772/4146 (18.6 %) | 126/725 (17.4 %) | 0.43 |
| Intake of statin (n/N, %) | 1341/4083 (32.8 %) | 222/710 (31.3 %) | 0.41 |
| **Comorbidities** | |  |  |
| Atrial fibrillation (n/N, %) | 1682/4164 (40.4 %) | 307/733 (41.9 %) | 0.45 |
| Previous Stroke (n/N, %) | 842/4166 (20.2 %) | 152/733 (20.7 %) | 0.74 |
| Coronary artery disease (n/N, %) | 1019/4148 (24.6 %) | 178/729 (24.4 %) | 0.93 |
| Peripheral artery disease (n/N, %) | 293/4107 (7.1 %) | 61/723 (8.4 %) | 0.22 |
| Arterial hypertension (n/N, %) | 3103/4174 (74.3 %) | 533/734 (72.6 %) | 0.33 |
| Diabetes mellitus (n/N, %) | 969/4176 (23.2 %) | 152/735 (20.7 %) | 0.13 |
| Hypercholesterolemia (n/N, %) | 1552/4143 (37.5 %) | 261/726 (36.0 %) | 0.44 |
| Active smoker (n/N, %) | 571/4066 (14.0 %) | 109/714 (14.7 %) | 0.39 |

mRS = modified Rankin Scale. NIHSS = National Institutes of Health Stroke Scale. CT = computed tomography. ASPECTS = Alberta Stroke Program Early Computed Tomography Score. IVT = Intravenous Thrombolytic Therapy. EVT = Endovascular Stroke Therapy. Log_2_(EASIX) = log2-transformed Endothelial Activation and Stress Index.

**Supplementary Table S2: Functional outcome and short-term mortality** (derivation cohort)
The 3-month survival rate was significantly better among the excluded patients, probably because patients with better neurological outcomes are generally more mobile, move more frequently, and therefore could not be followed up in the long term because the report data query was unsuccessful. Differences between the groups were examined through Chi square and Mann Whitney U tests, respectively. P values < 0.05 were considered significant.

| **Outcome** | **Patients included in final analyses** | **Patients where laboratory values crucial for EASIX or long-term survival data were missing** | **P Value** |
| --- | --- | --- | --- |
| mRS at 3-month, median (IQR) | 3 (1-5) | 3 (2-4) | 0.21 |
| **Death** | | | |
| in house (n/N, %) | 512/4176 (12.3 %) | 75/739 (10.1 %) | 0.11 |
| at 3-month (n/N, %) | 907/4131 (22.0 %) | 102/715 (14.3 %) | < 0.01 |

mRS = modified Rankin Scale. IQR = interquartile range

**Supplementary Table S3: Multivariable Cox regression analysis, full model** (derivation cohort, endpoint all-cause mortality)
Of 23 variables included in the full model, 14 showed significant impact on hazard of death and were consecutively included in the restricted model. P values < 0.05 were considered significant.

|  | **Hazard Ratio** | **95 % CI** | **P value** |
| --- | --- | --- | --- |
| Sex (cat) | 1.296 | 1.139 – 1.475 | < 0.01 |
| Age | 1.058 | 1.050 – 1.066 | < 0.01 |
| Pre-stroke mRS | 1.282 | 1.220 – 1.347 | < 0.01 |
| NIHSS on admission | 1.036 | 1.026 – 1.045 | < 0.01 |
| ASPECTS | 0.897 | 0.864 – 0.932 | < 0.01 |
| Systolic blood pressure | 1.001 | 0.999 – 1.003 | 0.259 |
| Diastolic blood pressure | 1.001 | 0.999 – 1.003 | 0.370 |
| Performance of IVT (cat) | 0.806 | 0.697 – 0.933 | < 0.01 |
| Performance of EVT (cat) | 1.067 | 0.888 – 1.281 | 0.488 |
| Previous stroke (cat) | 1.160 | 1.003 – 1.343 | 0.046 |
| Previous CAD (cat) | 0.965 | 0.832 – 1.119 | 0.637 |
| Previous hypertension (cat) | 0.920 | 0.781 – 1.083 | 0.315 |
| Previous PAD (cat) | 1.253 | 0.999 – 1.573 | 0.051 |
| Previous diabetes (cat) | 1.382 | 1.203 – 1.588 | < 0.01 |
| Previous hypercholesterinemia (cat) | 1.056 | 0.870 – 1.282 | 0.584 |
| Atrial fibrillation (cat) | 0.987 | 0.861 – 1.132 | 0.854 |
| Active smoking (cat) | 1.189 | 0.956 – 1.479 | 0.120 |
| Anticoagulant pretreatment (cat) | 1.302 | 1.095 – 1.548 | < 0.01 |
|  | **Hazard Ratio** | **95 % CI** | **P value** |
| Antiplatelet pretreatment (cat) | 1.188 | 1.026 – 1.376 | 0.021 |
| Statin pretreatment (cat) | 0.789 | 0.648 – 0.961 | 0.019 |
| C-reactive protein | 1.005 | 1.003 – 1.006 | < 0.01 |
| White blood cell count | 1.024 | 1.011 – 1.038 | < 0.01 |
| Log_2_(EASIX) | 1.202 | 1.115 – 1.296 | < 0.01 |

Cat = categorical variable. CI = confidence interval. mRS = modified Rankin Scale. NIHSS = National Institutes of Health Stroke Scale. ASPECTS = Alberta Stroke Program Early Computed Tomography Score. IVT = Intravenous Thrombolytic Therapy. EVT = Endovascular Stroke Therapy. CAD = coronary artery disease. PAD = peripheral artery disease. Log_2_(EASIX) = log2-transformed Endothelial Activation and Stress Index.

**Supplementary Table S4: Collinearity analysis of the variables of the multivariable (full) model** (derivation cohort).
All predictors have Variance Inflation Factor (VIF) values below 5, indicating low multicollinearity and stable, reliable coefficient estimates in the regression model.

|  | **Tolerance** | **Variance inflation factor** |
| --- | --- | --- |
| Sex | 0.862 | 1.160 |
| Age | 0.580 | 1.723 |
| Pre-stroke mRS | 0.707 | 1.415 |
| NIHSS on admission | 0.642 | 1.558 |
| ASPECTS | 0.792 | 1.262 |
| Systolic blood pressure | 0.916 | 1.092 |
| Diastolic blood pressure | 0.937 | 1.067 |
| Performance of IVT | 0.663 | 1.509 |
| Performance of EVT (cat) | 0.595 | 1.680 |
| Previous stroke (cat) | 0.841 | 1.189 |
| Previous CAD (cat) | 0.723 | 1.384 |
| Previous hypertension (cat) | 0.807 | 1.240 |
| Previous PAD (cat) | 0.935 | 1.069 |
| Previous diabetes (cat) | 0.916 | 1.091 |
| Previous hypercholesterinemia (cat) | 0.314 | 3.180 |
| Atrial fibrillation (cat) | 0.707 | 1.415 |
| Active smoking (cat) | 0.860 | 1.163 |
| Anticoagulant pretreatment (cat) | 0.629 | 1.591 |
|  | **Tolerance** | **Variance inflation factor** |
| Antiplatelet pretreatment (cat) | 0.651 | 1.536 |
| Statin pretreatment (cat) | 0.315 | 3.171 |
| C-reactive protein | 0.895 | 1.118 |
| White blood cell count | 0.912 | 1.096 |
| Log_2_(EASIX) | 0.826 | 1.210 |

Cat = categorical variable. CI = confidence interval. mRS = modified Rankin Scale. NIHSS = National Institutes of Health Stroke Scale. ASPECTS = Alberta Stroke Program Early Computed Tomography Score. IVT = Intravenous Thrombolytic Therapy. EVT = Endovascular Stroke Therapy. CAD = coronary artery disease. PAD = peripheral artery disease. Log_2_(EASIX) = log2-transformed Endothelial Activation and Stress Index.

**Supplementary Table S5: Multivariable Cox regression analysis, restricted model** (derivation cohort, endpoint all-cause mortality)
The restricted model includes the 14 variables significant in the full model. P values < 0.05 were considered significant.

|  | **Hazard ratio** | **95% CI** | **P value** |
| --- | --- | --- | --- |
| Sex (cat) | 1.297 | 1.159 – 1.452 | < 0.01 |
| Age | 1.051 | 1.044 – 1.057 | < 0.01 |
| Pre-stroke mRS | 1.263 | 1.209 – 1.319 | < 0.01 |
| NIHSS on admission | 1.041 | 1.033 – 1.049 | < 0.01 |
| ASPECTS | 0.899 | 0.870 – 0.928 | < 0.01 |
| Performance of IVT (cat) | 0.774 | 0.689 – 0.870 | < 0.01 |
| Previous stroke (cat) | 1.166 | 1.026 – 1.324 | 0.019 |
| Previous diabetes (cat) | 1.418 | 1.257 – 1.599 | < 0.01 |
| Anticoagulant pretreatment (cat) | 1.215 | 1.052 – 1.404 | < 0.01 |
| Antiplatelet pretreatment (cat) | 1.218 | 1.075 – 1.381 | < 0.01 |
| Statin pretreatment (cat) | 0.869 | 0.771 – 0.980 | 0.022 |
| C-reactive protein | 1.005 | 1.003 – 1.006 | < 0.01 |
| White blood cell count | 1.027 | 1.015 – 1.039 | < 0.01 |
| Log_2_(EASIX) | 1.201 | 1.124 – 1.282 | < 0.01 |

Cat = categorical variable. CI = confidence interval. mRS = modified Rankin Scale. NIHSS = National Institutes of Health Stroke Scale. ASPECTS = Alberta Stroke Program Early Computed Tomography Score. IVT = Intravenous Thrombolytic Therapy. Log_2_(EASIX) = log2-transformed Endothelial Activation and Stress Index.

**Supplementary Table S6: Subgroup analysis of the association between log_2_EASIX and mortality in acute ischemic stroke patients** (derivation cohort, Cox proportional hazard regression, restricted model, endpoint all-cause mortality).
The trend of the effect size was consistent in all subgroups. Hazard ratio and 95 % CI are shown. Significant interactions were found for age (p = 0.005) and presence of large vessel occlusion (p = 0.04).

|  | **Events (%)** | **HR (95 % CI)** | **P value for interaction** |
| --- | --- | --- | --- |
| Age <70 | 22.2 % | 1.47 (1.26–1.71) | < 0.01 |
| Age >= 70 | 54.3 % | 1.16 (0.92–1.45) |  |
| Infarct size <1/3 | 35.5 % | 1.20 (1.11–1.30) | 0.477 |
| Infarct size >=1/3 | 67.4 % | 1.26 (1.07–1.47) |  |
| NIHSS < 15 | 32.4 % | 1.25 (1.13–1.38) | 0.305 |
| NIHSS >=15 | 60.0 % | 1.17 (0.99–1.37) |  |
| THRIVE score <6 | 34.0 % | 1.22 (1.12–1.33) | 0.531 |
| THRIVE score >=6 | 70.0 % | 1.17 (1.01–1.37) |  |
| 2015-2019 | 52.8 % | 1.27 (1.15–1.39) | 0.105 |
| 2020-2024 | 36.5 % | 1.14 (0.98–1.34) |  |
| Pre-stroke mRS < 3 | 36.3 % | 1.25 (1.16–1.35) | 0.068 |
| Pre-stroke mRS >= 3 | 71.6 % | 1.10 (0.95–1.29) |  |
| ASPECTS 6-10 | 41.8 % | 1.19 (0.86–1.65) | 0.444 |
| ASPECTS 0-5 | 51.0 % | 1.31 (1.04–1.64) |  |
| No IVT | 53.3 % | 1.24 (1.14–1.36) | 0.266 |
| IVT | 37.6 % | 1.16 (0.99–1.35) |  |
| No diabetes | 40.2 % | 1.17 (1.08–1.26) | 0.172 |
| Diabetes | 55.1 % | 1.28 (1.09–1.50) |  |
|  | **Events (%)** | **HR (95 % CI)** | **P value for interaction** |
| No CAD | 39.7 % | 1.23 (1.14–1.34) | 0.256 |
| CAD | 55.6 % | 1.14 (0.97–1.33) |  |
| No LVO | 24.9 % | 1.22 (1.14–1.31) | 0.040 |
| LVO | 47.5 % | 1.21 (1.12–1.29) |  |

LVO = large vessel occlusion. CAD = coronary artery disease. IVT = intravenous thrombolytic therapy. ASPECTS = Alberta Stroke Program Early Computed Tomography Score. mRS = modified Rankin Scale. THRIVE = Totaled Health Risks in Vascular Events. NIHSS = National Institutes of Health Stroke Scale.

**Supplementary Table S7: All three EASIX components correlate significantly with the endpoint all-cause mortality in a separate multivariable Cox proportional regression model** (derivation cohort, endpoint all-cause mortality)
This demonstrates the prognostic value of the single EASIX components. P values < 0.05 were considered significant.

|  | **Hazard Ratio** | **95 % CI** | **P value** |
| --- | --- | --- | --- |
| LDH (log_2_) | 1.749 | 1.613 – 1.896 | < 0.01 |
| Creatinine (log_2_) | 1.528 | 1.392 – 1.678 | < 0.01 |
| Platelet count (log_2_) | 0.846 | 0.772 – 0.928 | < 0.01 |

EASIX = Endothelial Activation and Stress Index. LDH = lactate dehydrogenase.

**Supplementary Table S8: Multivariable binary logistic regression analysis** (endpoint 3-month mortality, derivation cohort). P values < 0.05 were considered significant.

|  | **Odds Ratio** | **95 % CI** | **P value** |
| --- | --- | --- | --- |
| Sex (cat) | 1.170 | 0.964 – 1.419 | 0.113 |
| Age | 1.038 | 1.027 – 1.049 | < 0.01 |
| Pre-stroke mRS | 1.287 | 1.196 – 1.385 | < 0.01 |
| NIHSS on admission | 1.088 | 1.075 – 1.101 | < 0.001 |
| Large vessel occlusion (cat) | 1.923 | 1.184 – 3.123 | < 0.01 |
| Performance of IVT (cat) | 0.748 | 0.604 – 0.927 | < 0.01 |
| Performance of EVT (cat) | 1.102 | 0.769 – 1.578 | 0.598 |
| Previous stroke (cat) | 1.425 | 1.136 – 1.788 | < 0.01 |
| Previous hypertension (cat) | 0.795 | 0.626 – 1.009 | 0.059 |
| Previous diabetes (cat) | 1.840 | 1.491 – 2.272 | < 0.01 |
| Previous hypercholesterinemia (cat) | 0.674 | 0.548 – 0.831 | < 0.01 |
| Atrial fibrillation (cat) | 0.969 | 0.783 – 1.199 | 0.772 |
| Active smoking (cat) | 1.061 | 0.765 – 1.470 | 0.724 |
| Anticoagulant pretreatment (cat) | 1.268 | 0.977 – 1.647 | 0.075 |
| Antiplatelet pretreatment (cat) | 1.310 | 1.051 – 1.632 | 0.016 |
| C-reactive protein | 1.007 | 1.004 – 1.009 | < 0.01 |
| White blood cell count | 1.070 | 1.044 – 1.097 | < 0.01 |
| Log_2_(EASIX) | 1.408 | 1.262 – 1.570 | < 0.01 |

Cat = categorical variable. CI = confidence interval. mRS = modified Rankin Scale. NIHSS = National Institutes of Health Stroke Scale. IVT = Intravenous Thrombolytic Therapy. EVT = Endovascular Stroke Therapy. CAD = coronary artery disease. PAD = peripheral artery disease. Log_2_(EASIX) = log2-transformed Endothelial Activation and Stress Index.

**Supplementary Table S9: Multivariable binary logistic regression analysis (validation cohort) with EASIX cut-off point 1.211** (endpoint 3-month survival)

EASIX predicts hazards of mortality with an Odds Ratio of 1.86 (95% CI: 1.28-2.70), p < 0.01 in this multivariable model in the validation cohort.

|  | **Odds Ratio** | **95% CI** | **P value** |
| --- | --- | --- | --- |
| Sex (cat) | 1.607 | 1.091 – 2.367 | 0.016 |
| Age | 1.086 | 1.063 – 1.110 | < 0.01 |
| Pre-stroke mRS | 1.335 | 1.180 – 1.510 | < 0.01 |
| NIHSS on admission | 1.154 | 1.119 – 1.190 | < 0.01 |
| Large vessel occlusion (cat) | 1.460 | 0.887 – 2.404 | 0.137 |
| Performance of IVT (cat) | 0.641 | 0.419 – 0.981 | 0.041 |
| Performance of EVT (cat) | 0.956 | 0.550– 1.664 | 0.875 |
| Previous stroke (cat) | 1.402 | 0.908 – 2.164 | 0.127 |
| Previous hypertension (cat) | 0.679 | 0.394 – 1.172 | 0.165 |
| Previous diabetes (cat) | 1.835 | 1.238 – 2.720 | < 0.01 |
| Previous hypercholesterinemia (cat) | 0.936 | 0.640 – 1.369 | 0.733 |
| Atrial fibrillation (cat) | 0.646 | 0.415 – 1.004 | 0.052 |
| Active smoking (cat) | 2.170 | 1.364 – 3.453 | < 0.01 |
| Anticoagulant pretreatment (cat) | 1.177 | 0.709 – 1.956 | 0.528 |
| Antiplatelet pretreatment (cat) | 0.542 | 0.351 – 0.838 | < 0.01 |
| C-reactive protein | 1.013 | 1.008 – 1.017 | < 0.01 |
| White blood cell count | 1.006 | 0.995 – 1.017 | 0.299 |
| EASIX >= 1.211 (cat) | 1.857 | 1.277 – 2.702 | < 0.01 |

Cat = categorical variable. CI = confidence interval. mRS = modified Rankin Scale. NIHSS = National Institutes of Health Stroke Scale. IVT = Intravenous Thrombolytic Therapy. EVT = Endovascular Stroke Therapy. CAD = coronary artery disease. PAD = peripheral artery disease. EASIX = Endothelial Activation and Stress Index.

**Supplementary Table S10:** **Validation of EASIX cut-off value 1.211 as an independent predictor of 3-month survival in acute ischemic stroke patients** (validation cohort adjusted to PI coefficients derived from the multivariable model of the derivation cohort).

The prognostic index with a multivariable model including binary EASIX (cut-off at 1.211) shows a lower Brier score and a higher C-statistic compared to a model without EASIX.

|  | **Prognostic Index (PI) with binary EASIX (Cut-Off 1.211)** | **PI without binary EASIX** |
| --- | --- | --- |
| **Brier score** | 0.0899 | 0.0956 |
| **C-statistic** | 0.867 | 0.857 |

EASIX: Endothelial activation and Stress Index. C-statistic: concordance statistic.

**Supplementary Table S11**: **Baseline characteristics in comparison between patients where 3-months outcome was missing and those where it was available** (validation cohort)
Differences between the groups were examined through Chi square and Mann Whitney U tests, respectively. P values < 0.05 were considered significant. Patients in which 3-month outcome was not available were overall younger, in a better premorbid condition, had less severe strokes reflected by lower NIHSS scores at admission and lower frequency of vessel occlusion in CT-angiography, were consequently less often subject to thrombectomy and showed lower EASIX values.

|  | **Patients with available 3-month outcome** | **Patients without available 3-month outcome** | **P value** |
| --- | --- | --- | --- |
| All patients, n | 1758 | 515 |  |
| **Demographics** | | | |
| Female sex (n/N, %) | 860/1758 (48.9 %) | 242/515 (47.0 %) | 0.44 |
| Age (median [IQR]) | 76 (65-83) [N = 1758) | 72(60-79) [N = 515] | < 0.01 |
| **Functional status** | | | |
| pre-stroke mRS (median [IQR]) | 0 (0-2) [N = 1758] | 0 (0-1) [N = 515] | 0.01 |
| NIHSS score at admission (median [IQR]) | 4 (2-9) [N = 1746] | 3 (1-7) [N = 513] | < 0.01 |
| **Acute stroke treatment** | | | |
| Vessel occlusion in CT-angiography (n/N, %) | 454/1758 (25.8 %) | 101/515 (19.6 %) | < 0.01 |
| IVT (n/N, %) | 516/1752 (29.5 %) | 148/513 (28.8 %) | 0.79 |
| EVT (n/N, %) | 268/1758 (15.2 %) | 55/514 (10.7 %) | 0.01 |
| EASIX (median [IQR]) | 0.845 (0.607-1.251) | 0.792 (0.553-1.179) | 0.01 |
|  | **Patients with available 3-month outcome** | **Patients without available 3-month outcome** | **P value** |
| **Prior medication** | | | |
| Mono platelet inhibition (n/N, %) | 542/1758 (30.8 %) | 137/515 (26.6 %) | 0.07 |
| Dual platelet inhibition (n/N, %) | 30/1758 (1.7 %) | 6/515 (1.2 %) | 0.39 |
| Anticoagulation (n/N, %) | 277/1758 (15.8 %) | 65/515 (12.6 %) | 0.08 |
| **Comorbidities** | | | |
| Atrial fibrillation (n/N, %) | 530/1758 (30.1 %) | 117/515 (22.7 %) | < 0.01 |
| Previous Stroke (n/N, %) | 366/1708 (21.4 %) | 96/496 (19.4 %) | 0.32 |
| Arterial hypertension (n/N, %) | 1518/1747 (86.9 %) | 419/512 (81.8 %) | < 0.01 |
| Diabetes mellitus (n/N, %) | 467/1738 (26.9 %) | 138/509 (27.1 %) | 0.91 |
| Hypercholesterolemia (n/N, %) | 1180/1704 (69.2 %) | 368/501 (73.5 %) | 0.07 |
| Active smoker (n/N, %) | 463/1664 (27.8 %) | 158/456 (34.6 %) | < 0.01 |

mRS = modified Rankin Scale. NIHSS = National Institutes of Health Stroke Scale. CT = computed tomography. ASPECTS = Alberta Stroke Program Early Computed Tomography Score. IVT = Intravenous Thrombolytic Therapy. EVT = Endovascular Stroke Therapy. EASIX = Endothelial Activation and Stress Index.

**Supplementary Table S12: Functional outcome and short-term mortality** (validation cohort)
Differences between the groups were examined through Chi square and Mann Whitney U tests, respectively. P values < 0.05 were considered significant.

|  | **Patients with available 3-month outcome** | **Patients without available 3-month outcome** | **P Value** |
| --- | --- | --- | --- |
| mRS at 3-month, median (IQR) | 2 (0-4) [N = 1758] | na | na |
| mRS at discharge, median (IQR) | 2 (1-4) [N = 1758] | 2 (0-4) [N = 515] | < 0.01 |
| **Death** | | | |
| in house (n/N, %) | 62/1758 (3.5 %) | 0/515 (0 %) | < 0.01 |
| at 3-month (n/N, %) | 292/1758 (16.6 %) | na | na |

mRS = modified Rankin Scale. IQR = interquartile range

**Supplementary Table S13:** **Best case analysis –** **Multivariable binary logistic regression analysis with EASIX cut-off point 1.211** (validation cohort; endpoint 3-month survival).
For all patients for whom no survival data were available at 3 months, survival was assumed.

EASIX predicts hazard of mortality with an Odds Ratio of 1.78 (95 % CI: 1.24-2.54), p < 0.01 in this multivariable model in the validation cohort. The model performs like the original model.

|  | **Odds Ratio** | **95% CI** | **P value** |
| --- | --- | --- | --- |
| Sex (cat) | 1.519 | 1.049 – 2.200 | 0.027 |
| Pre-stroke mRS | 1.274 | 1.134 – 1.433 | < 0.01 |
| NIHSS on admission | 1.139 | 1.107 – 1.172 | < 0.01 |
| Performance of IVT (cat) | 0.625 | 0.413 – 0.945 | 0.026 |
| Performance of EVT (cat) | 1.130 | 0.662 – 1.926 | 0.655 |
| Large vessel occlusion (cat) | 1.439 | 0.895 – 2.313 | 0.133 |
| Previous diabetes (cat) | 1.650 | 1.136 – 2.398 | < 0.01 |
| Previous stroke (cat) | 1.464 | 0.967 – 2.216 | 0.072 |
| Previous hypertension (cat) | 0.729 | 0.431 – 1.232 | 0.238 |
| Previous hypercholesterinemia (cat) | 0.902 | 0.625 – 1.300 | 0.579 |
| Active smoking (cat) | 1.980 | 1.273 – 3.079 | < 0.01 |
| Atrial fibrillation (cat) | 0.706 | 0.462 – 1.080 | 0.108 |
| Anticoagulant pretreatment (cat) | 1.189 | 0.726 – 1.946 | 0.492 |
| Antiplatelet pretreatment (cat) | 0.631 | 0.418 – 0.954 | 0.029 |
| C-reactive protein | 1.012 | 1.008 – 1.016 | < 0.01 |
|  | **Odds Ratio** | **95% CI** | **P value** |
| White blood cell count | 1.007 | 0.997 – 1.017 | 0.160 |
| Age | 1.084 | 1.062 – 1.106 | < 0.01 |
| EASIX >= 1.211 (cat) | 1.779 | 1.244 – 2.543 | < 0.01 |

Cat = categorical variable. CI = confidence interval. mRS = modified Rankin Scale. NIHSS = National Institutes of Health Stroke Scale. IVT = Intravenous Thrombolytic Therapy. EVT = Endovascular Stroke Therapy. CAD = coronary artery disease. PAD = peripheral artery disease. Log_2_(EASIX) = log2-transformed Endothelial Activation and Stress Index.

**Supplementary Table S14: Worst case analysis –** **Multivariable binary logistic regression analysis with EASIX cut-off point 1.211** (validation cohort; endpoint 3-month survival).
For all patients for whom no survival data were available at 3 months, death was assumed.

EASIX predicts hazard of mortality with an Odds Ratio of 1.28 (95 % CI: 1.02-1.61), p = 0.034 in this multivariable model in the validation cohort.

|  | **Odds Ratio** | **95 % CI** | **P value** |
| --- | --- | --- | --- |
| Sex (cat) | 0.971 | 0.793 – 1.189 | 0.774 |
| Pre-stroke mRS | 1.169 | 1.080 – 1.265 | < 0.01 |
| NIHSS on admission | 1.058 | 1.037 – 1.079 | < 0.01 |
| Performance of IVT (cat) | 0.834 | 0.661 – 1.052 | 0.126 |
| Performance of EVT (cat) | 0.704 | 0.482 – 1.026 | 0.068 |
| Large vessel occlusion (cat) | 1.113 | 0.822 – 1.507 | 0.488 |
| Previous diabetes (cat) | 1.182 | 0.947 – 1.474 | 0.138 |
| Previous stroke (cat) | 1.018 | 0.785 – 1.320 | 0.893 |
| Previous hypertension (cat) | 0.719 | 0.541 – 0.954 | 0.022 |
| Previous hypercholesterinemia (cat) | 1.058 | 0.851 – 1.316 | 0.611 |
| Active smoking (cat) | 1.214 | 0.964 – 1.527 | 0.099 |
| Atrial fibrillation (cat) | 0.850 | 0.650 – 1.111 | 0.233 |
|  | **Odds Ratio** | **95 % CI** | **P value** |
| Anticoagulant pretreatment (cat) | 0.955 | 0.685 – 1.332 | 0.787 |
| Antiplatelet pretreatment (cat) | 0.757 | 0.598 – 0.958 | 0.021 |
| C-reactive protein | 1.006 | 1.002 – 1.009 | < 0.01 |
| White blood cell count | 0.993 | 0.971 – 1.016 | 0.551 |
| Age | 1.002 | 0.993 – 1.011 | 0.637 |
| EASIX >= 1.211 (cat) | 1.279 | 1.018 – 1.606 | 0.034 |

Cat = categorical variable. CI = confidence interval. mRS = modified Rankin Scale. NIHSS = National Institutes of Health Stroke Scale. IVT = Intravenous Thrombolytic Therapy. EVT = Endovascular Stroke Therapy. CAD = coronary artery disease. PAD = peripheral artery disease. Log_2_(EASIX) = log2-transformed Endothelial Activation and Stress Index.

**Supplementary Table S15: Best/worst case analysis –** **Validation of EASIX cut-off value 1.211 as an independent predictor of 3-month survival in acute ischemic stroke patients** (validation cohort adjusted to PI coefficients derived from the multivariable model of the derivation cohort).

| **Validation cohort – Best case analysis** | **Prognostic Index (PI) with binary EASIX (Cut-Off 1.211)** | **PI without binary EASIX** |
| --- | --- | --- |
| **Brier score** | 0.0734 | 0.0769 |
| **C-statistic** | 0.872 | 0.862 |
| **Validation cohort – Worst case analysis** | **Prognostic Index (PI) with binary EASIX (Cut-Off 1.211)** | **PI without binary EASIX** |
| **Brier score** | 0.2577 | 0.2722 |
| **C-statistic** | 0.588 | 0.585 |

EASIX: Endothelial Activation and Stress Index. C-statistic: concordance statistic.

**Supplementary Table S16: Binary EASIX (Cut-Off 1.211) provides added value when taken together with the THRIVE score, a known prognostic score in acute ischemic stroke.** Notably, the THRIVE score has originally been developed to predict mortality and functional outcome in patients receiving reperfusion therapies. Nevertheless, the predictive performance seems even better in the validation cohort which is not restricted to patients receiving reperfusion therapies.

| **Derivation cohort** | **THRIVE components with binary EASIX (Cut-Off 1.211)** | **THRIVE components without binary EASIX** |
| --- | --- | --- |
| C-statistic | 0.775 | 0.765 |
| **Validation cohort** | **THRIVE components with binary EASIX (Cut-Off 1.211)** | **THRIVE components without binary EASIX** |
| C-statistic | 0.816 | 0.802 |

THRIVE = Totaled health risks in vascular events. EASIX: Endothelial Activation and Stress Index. C-statistic: concordance statistic.

**Supplementary Table S17:** **Patient characteristics - group comparison between patients receiving IVT or EVT** (derivation cohort).

Patients receiving IVT alone showed a substantially lower NIHSS (median 5 vs 15) and lower rate of vessel occlusions (36.8 % vs 99.8 %) than patients receiving EVT with or without IVT.

|  | **IVT alone** | **EVT (+/- IVT)** |
| --- | --- | --- |
| All patients, n | 1142 | 3046 |
| **Demographics** | | |
| Female sex (n/N, %) | 524/1142 (45.9 %) | 1622/3046 (53.3 %) |
| Age (median [IQR] | 75 (65-82) [N = 1142] | 77 (66-84) [N = 3046) |
| **Functional status** | | |
| pre-stroke mRS (median [IQR]) | 0 (0-2) [N = 1134] | 0 (0-2) [N = 3016] |
| NIHSS score at admission (median [IQR]) | 5 (3-9) [N = 1140] | 15 (9-20) [N = 3032] |
| **Acute stroke treatment** | | |
| Vessel occlusion in CT-angiography (n/N, %) | 407/1107 (36.8 %) | 3039/3045 (99.8 %) |
| ASPECTS (median [IQR]) | 10 (10-10) [N = 950] | 9 (8-10) [N = 2566] |
| Systolic blood pressure (median [IQR]) | 160 (146-176) [N = 1079] | 159 (140-175) [N = 2585] |
| IVT (n/N, %) | 1142/1142 (100 %) | 1405/3046 (46.1 %) |
| EVT total (n/N, %) | 0/1142 (0 %) | 3046/3046 (100.0 %) |
| - Intracranial EVT (n/N, %) |  | 2709/3036 (89.2 %) |
| - Extracranial EVT (n/N, %) |  | 495/3046 (16.3 %) |
| **Prior medication** | | |
| Mono platelet inhibition (n/N, %) | 413/1138 (36.3 %) | 830/2992 (27.7 %) |
| Dual platelet inhibition (n/N, %) | 35/1138 (3.1 %) | 83/2992 (2.8 %) |
| Anticoagulation (n/N, %) | 65/1136 (5.7 %) | 707/3010 (23.5 %) |
| Intake of statin (n/N, %) | 362/1132 (32.0 %) | 979/2951 (33.2 %) |
|  | **IVT alone** | **EVT (+/- IVT)** |
| **Comorbidities** | | |
| Atrial fibrillation (n/N, %) | 248/1137 (21.8 %) | 1434/3027 (47.4 %) |
| Previous Stroke (n/N, %) | 239/1140 (21.0 %) | 603/3026 (19.9 %) |
| Coronary artery disease (n/N, %) | 254/1134 (22.4 %) | 765/3014 (25.4 %) |
| Peripheral artery disease (n/N, %) | 62/1128 (5.5 %) | 231/2979 (7.8 %) |
| Arterial hypertension (n/N, %) | 839/1140 (73.6 %) | 2264/3034 (74.6 %) |
| Diabetes mellitus (n/N, %) | 251/1141 (22.0 %) | 718/3035 (23.7 %) |
| Hypercholesterolemia (n/N, %) | 422/1140 (37.0 %) | 1130/3003 (37.6 %) |
| Active smoker (n/N, %) | 185/1124 (16.5 %) | 386/2942 (13.1 %) |

mRS = modified Rankin Scale. NIHSS = National Institutes of Health Stroke Scale. ASPECTS = Alberta Stroke Program Early Computed Tomography Score. EVT = Endovascular Stroke Therapy. IVT = Intravenous Thrombolytic Therapy.

|  | **Derivation cohort**  Cut-off 1.211 | **Validation cohort**  Cut-off 1.211 | **Validation cohort**  Cut-off 2.32 |
| --- | --- | --- | --- |
| Sensitivity | 45.6% | 44.2% | 17.1% |
| Specificity | 72.1% | 76.9% | 94.4% |
| Positive predictive value | 31.5% | 27.6% | 40.3% |
| Negative predictive value | 82.5% | 93.4% | 85.2% |

**Supplementary Table S18:** **Further performance metrics of binary EASIX in the prediction of 3-months mortality.**

**Supplementary Table S19: Validation of EASIX cut-off value 2.32 as an independent predictor of 3-month survival in acute ischemic stroke patients** (validation cohort adjusted to PI coefficients derived from the multivariable model of the derivation cohort).

The prognostic index with a multivariable model including binary EASIX (cut-off at 2.32) shows a lower Brier score and a higher C-statistic compared to a model without EASIX.

| **Validation cohort** | **Prognostic Index (PI) with binary EASIX (cut-off 2.32)** | **PI without binary EASIX** |
| --- | --- | --- |
| **Brier score** | 0.0880 | 0.0956 |
| **C-statistic** | 0.866 | 0.857 |

**Supplementary Figure S1: Flowchart of patient inclusion/exclusion in the derivation cohort.**Of 5,213 patients eligible, 4,188 were included in final analyses.

**5,213** patients with acute reperfusion therapy for suspected acute ischemic stroke

**135** no ischemic stroke
**95** recurrent events
**40** hemodialysis dependence **3** age < 18 years

**4,940** patients further explored

**629** survival data unavailable
**123** EASIX components missing

**4,188** patients included in final analyses

**Supplementary Figure S2: Flowchart of patient inclusion/exclusion in the validation cohort.**Of 2,375 patients eligible, 2,273 were included in final analyses.

**2,375** patients with acute ischemic stroke

**2** age < 18 years

**2,373** patients further explored

**100** EASIX components missing

**2,273** patients included in final analyses

**Supplementary Figure S3: ROC analysis (PI – EASIX only) in the derivation** **and validation cohort.**

**
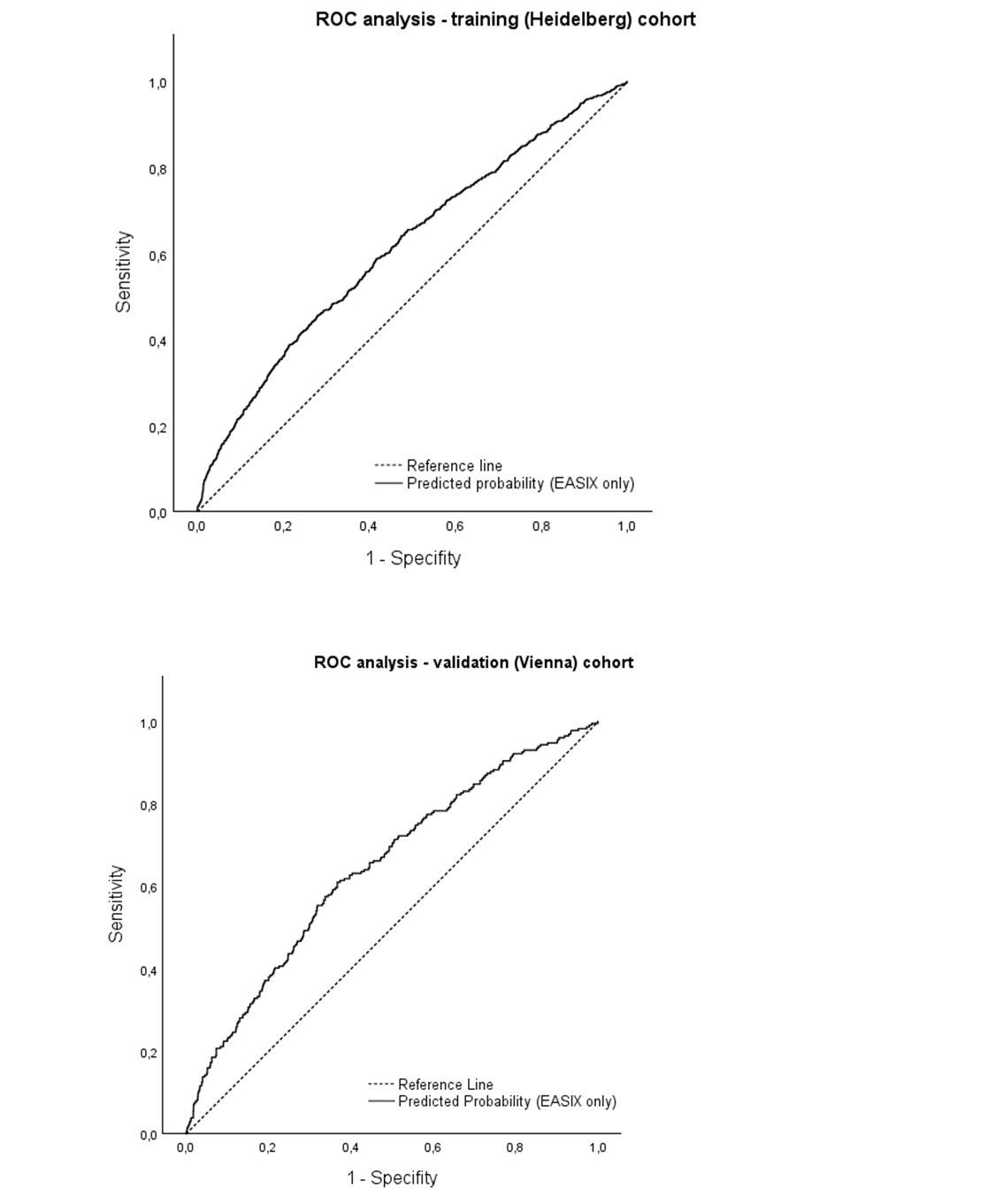
**

ROC = Receiver Operating Characteristics curve. PI = Prognostic index. EASIX = Endothelial Activation and Stress Index.

**Supplementary Figure S4: Maximum Youden Index (Sensitivity + Specificity – 1) was found at EASIX = 1.211.**


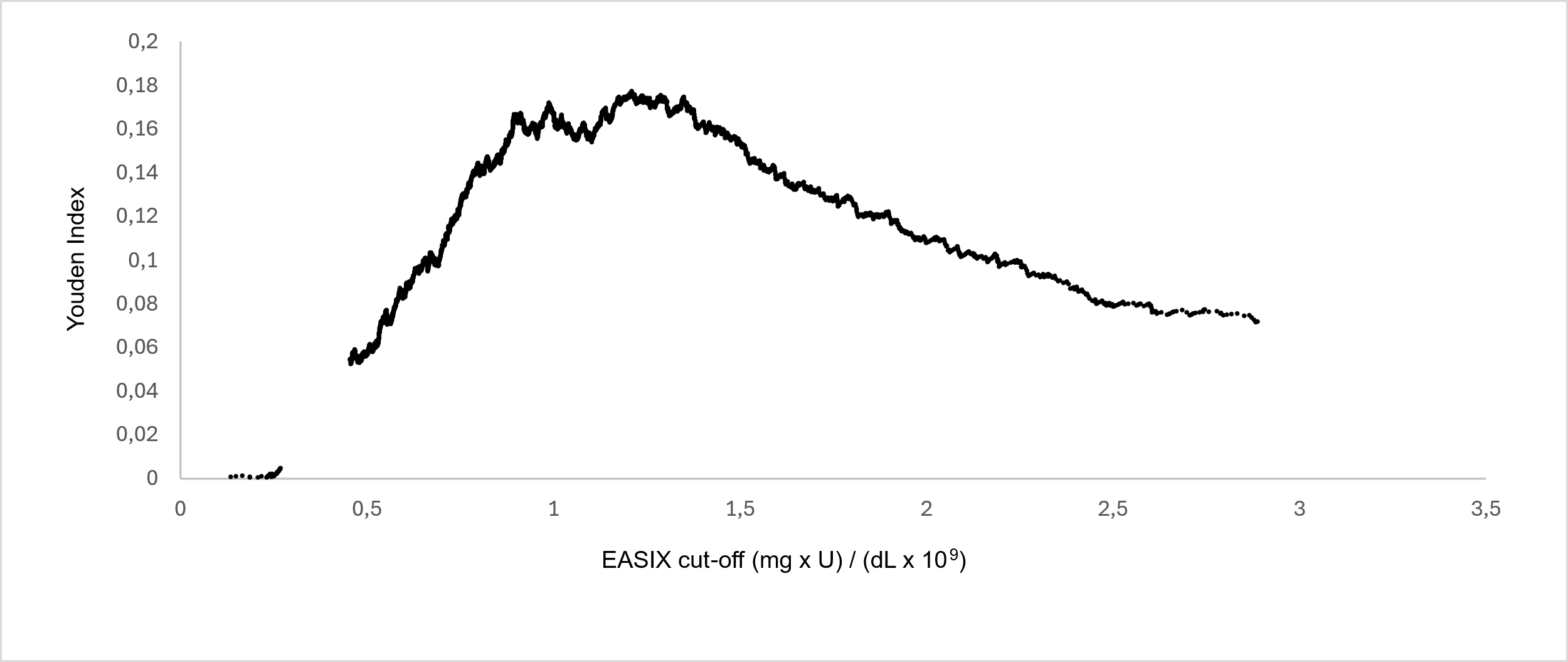


EASIX = Endothelial Activation and Stress Index.

**Supplementary Figure S5: Functional outcome after 3 months was better in patients with lower EASIX in the derivation cohort.**EASIX < 0.88 is the third IQR of n = 47 healthy individuals included in the “Endothelial Cell Dysfunction and Outcome (EndoCDO-H)” study, ^36^ validated in a cohort of patients with CAD. ^25^ Functional outcome is depicted by modified Rankin scale (mRS), ranging from 0 (no disabling deficit) to 6 (death).

**
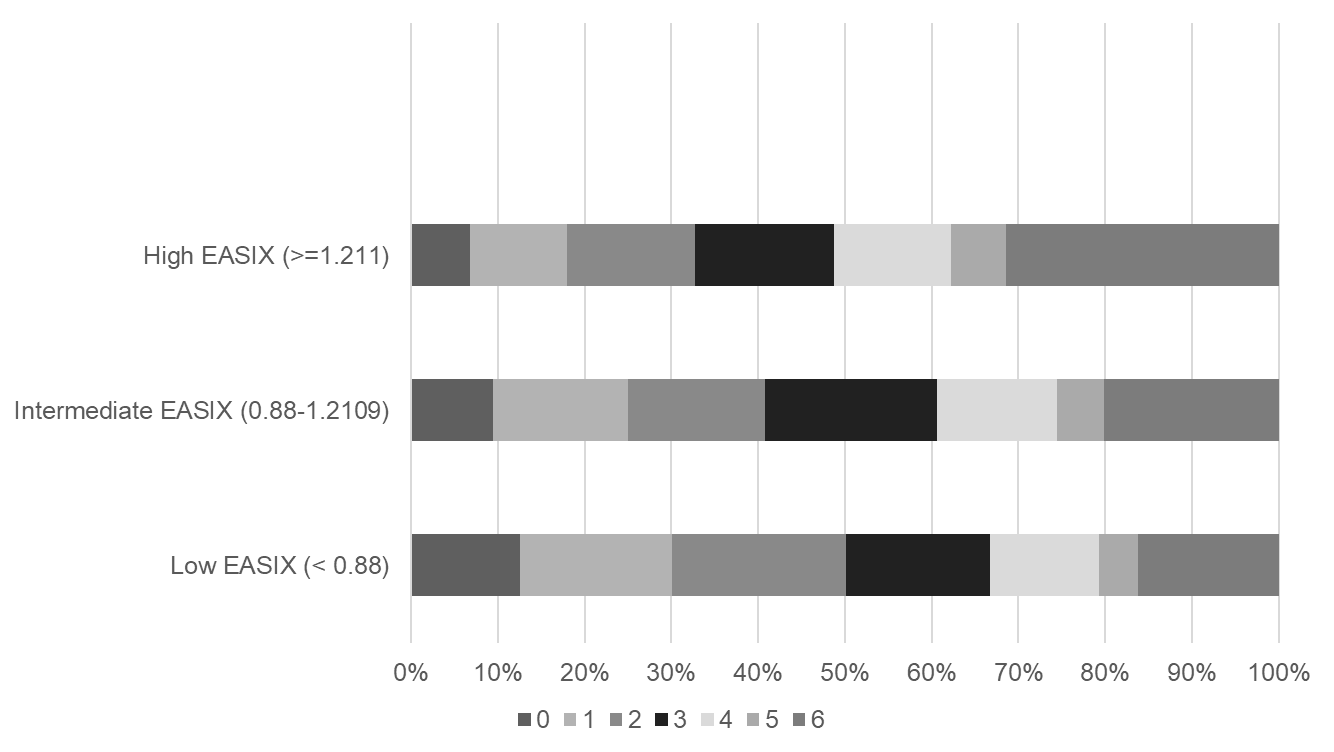
**

EASIX = Endothelial Activation and Stress Index. IQR = interquartile range. CAD = coronary artery disease.

**Supplementary Figure S6: Exploratory subgroup analyses for different ischemic stroke subtypes** (derivation cohort, restricted model).

Event rates: Large-artery atherosclerosis: 1400 events, cardioembolic stroke 746 events, small-vessel disease: 33 events, stroke of other determined cause: 27 events, stroke of undetermined cause: 325 events.


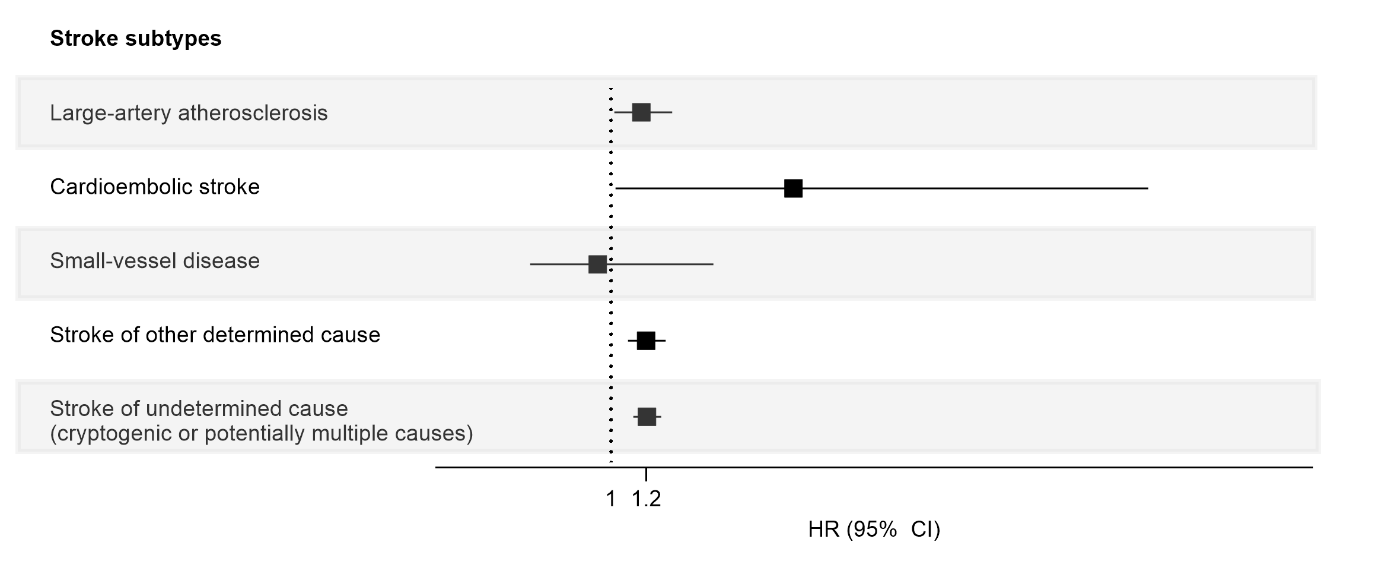

Supplement: Supplementary file 1 — Supplementary file1 (DOCX 15610 KB) [file 415_2026_13941_MOESM1_ESM.docx]
